# Supplementary material for: Neutrophils promote the development of reparative macrophages mediated by ROS to orchestrate liver repair
Source: Nat Commun. 2019 Mar 6;10:1076. doi: 10.1038/s41467-019-09046-8 (PMC6403250; doi:10.1038/s41467-019-09046-8)
Supplement: Supplementary file 1 — Supplementary Information [file 41467_2019_9046_MOESM1_ESM.pdf]

Supplementary Information

Neutrophils promote the development of reparative macrophages mediated by ROS to orchestrate liver repair

Wenting Yang et al

Supplementary Figure 1

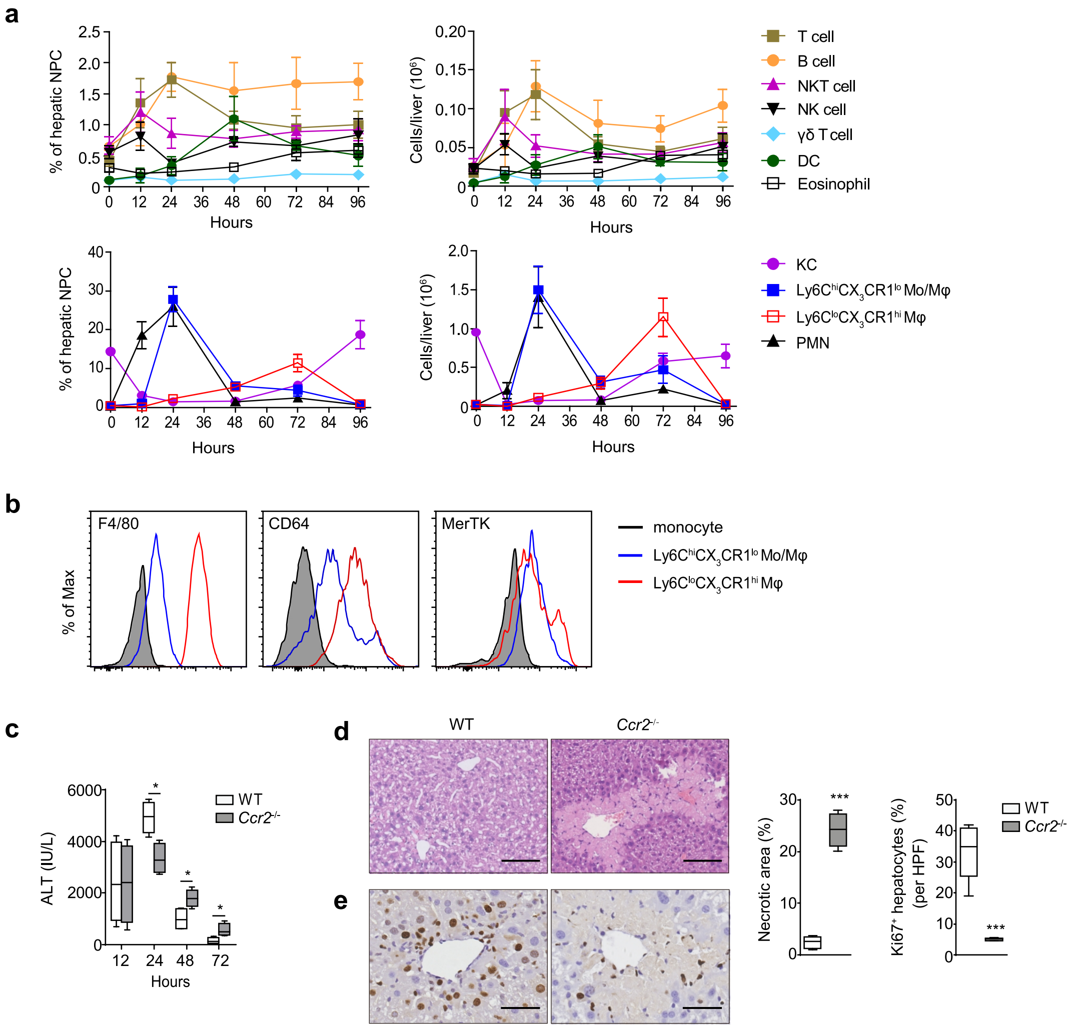

Supplementary Figure 1. Depletion of monocyte/macrophages impairs liver repair.

(**a**) C57BL/6 mice were challenged with APAP (400 mg kg^{-1}). The percentage and absolute numbers of the immune cell repertoire in the livers at each time point were determined by flow cytometry. Liver non-parenchymal cells (NPCs) were identified by first gating for live CD45$^+$ leukocytes. KC: Kupffer cells; Mo/Mφ: monocyte/macrophage; PMN: neutrophil. n=3. Experiment was repeated three times. (**b**) Flow cytometric analysis of key macrophage markers (F4/80, CD64 and MerTK) on blood monocytes, 24 hr Ly6C^{hi}CX$_3$CR1^{lo} cells and 72 hr Ly6C^{lo}CX$_3$CR1^{hi} cells. Experiment was repeated three times. (**c**-**e**) WT and *Ccr2*$^{-/-}$ mice were challenged with APAP. Serum alanine aminotransferase (ALT) levels at each time point (**c**), representative images of Hematoxylin and eosin stain (H&E) staining (**d**) and Immunohistochemistry (IHC) staining for Ki67 (**e**) in liver sections at 72 hr after APAP challenge are shown. Scale bars, 100 μm (**d**), 50 μm (**e**). n=5, 4. Experiment was repeated three times. Whiskers show min to max. Bars show the median (*P<0.05, ***P<0.001). P values were calculated by two-tailed Student's t-test (**c**-**e**).

Supplementary Figure 2

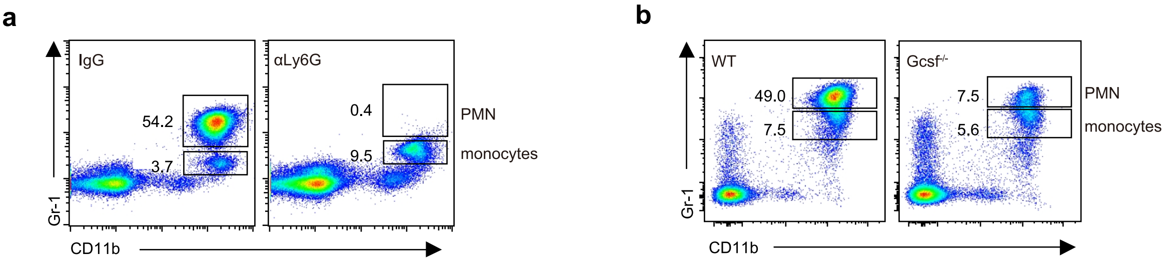

Supplementary Figure 2. Depletion of neutrophils using anti-Ly6G antibodies or Gcsf$^{-/-}$ mice.

(**a**) Mice were treated with the anti-Ly6G mAb or IgG at 6 hr after APAP challenge. Representative dot plots of blood neutrophils gated on CD45^{+} cells at 24 hr. The Gr-1 antibody was used for this case to avoid false-negative results since the anti-Ly6G depleting antibody could mask the Ly6G epitope. Experiment was repeated twice. (**b**) Representative dot plots of blood neutrophils gated on CD45^{+} cells in WT and Gcsf$^{-/-}$ mice at 24 hr. Experiment was repeated twice.

Supplementary Figure 3

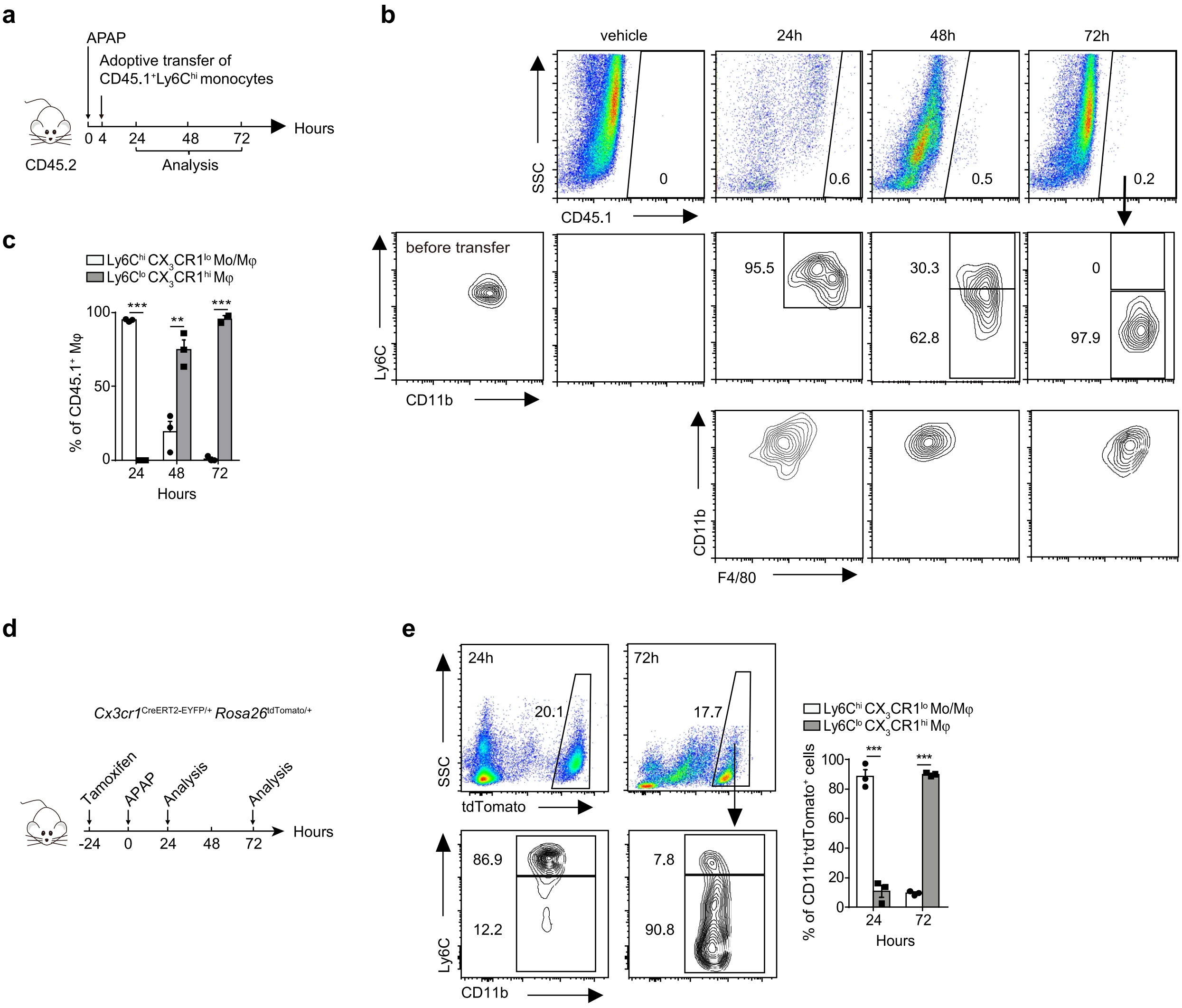

Supplementary Figure 3. Ly6C^{lo}CX$_3$CR1^{hi} macrophages derive from recruited Ly6C^{hi}CX$_3$CR1^{lo} monocytes/ macrophages.

(**a**) Schematic of the experimental design. CD115$^+$Ly6C^{hi} monocytes enriched with CD45.1 bone marrow cells were adoptively transferred into CD45.2 mice 4 hr after APAP challenge. (**b-c**) Identification of the transferred CD45.1$^+$ monocytes (gating on CD45.2$^-$) in the livers harvested at the indicated time points (**b**). Quantification of the percentage of identified CD45.1$^+$ hepatic macrophages that formed each of the macrophage subsets (**c**). n=3. Experiment was repeated three times. (**d-e**) Schematic of the experimental design (**d**). *Cx3cr1*$^{CreERT2\text{-}EYFP/+}$*Rosa26*$^{tdTomato/+}$ mice were given tamoxifen via oral gavage one day before APAP challenge. Representative flow cytometry analysis of Ly6C expression on CD11b$^+$tdTomato$^+$ cells in the livers of *Cx3cr1*$^{CreERT2\text{-}EYFP/+}$*Rosa26*$^{tdTomato/+}$ mice at 24 hr and 72 hr post APAP challenge (**e**). n=3. Experiment was repeated twice. The data shown are mean ± s.e.m. (**P<0.01, ***P<0.001). P values were calculated by two-tailed Student's t-test (**c**, **e**).

Supplementary Figure 4

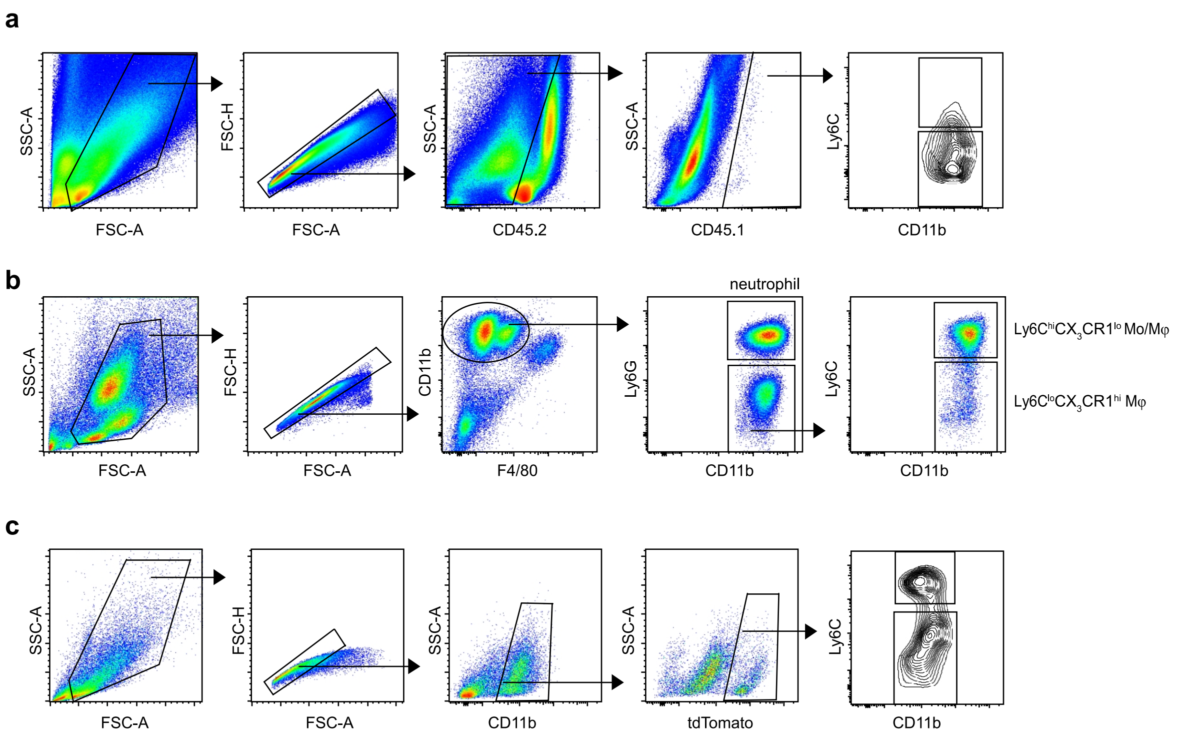

Supplementary Figure 4. Gating strategies used for FACS analysis.

(**a**) Gating strategy to determine the transferred CD45.1^{+} monocytes in the livers presented on **Fig. 2d**. (**b**) Gating strategy to sort hepatic neutrophils and macrophage subsets for in vitro co-cultures presented on **Fig. 2e-h** and **5a-c**. (**c**) Gating strategy to determine tdTomato^{+} cells in the livers presented on **Fig. 3j**.

Supplementary Figure 5

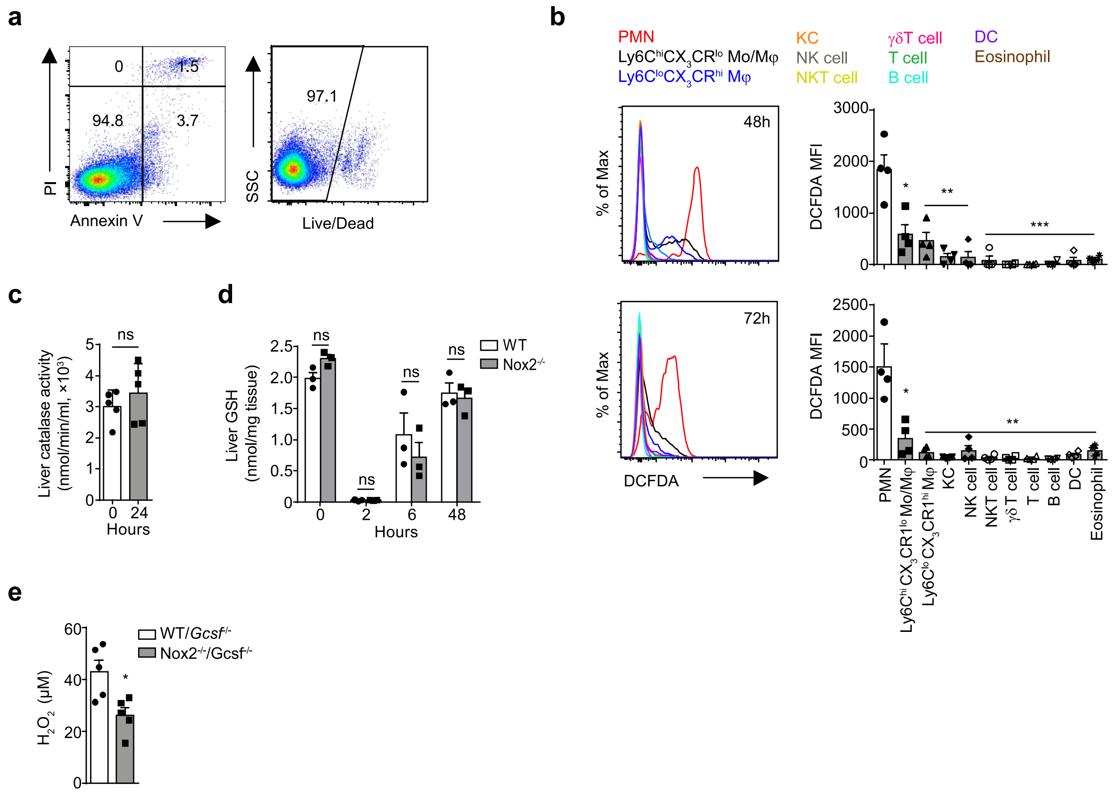

Supplementary Figure 5. Neutrophils are the primary source of ROS.

(**a**) Viability of purified 24 hr hepatic neutrophils was assessed before co-culture with macrophages. Staining of neutrophils with Annexin-V and propidium iodide (PI), or fixable viability dye eFluor 450. Experiment was repeated twice. (**b**) Flow cytometric analysis and quantification of the MFI of CM-H_2DCFDA in the various leukocytes isolated from livers at 48 hr and 72 hr after APAP challenge. n=4. Experiment was repeated three times. (**c**) The levels of catalase in liver homogenates from normal and 24 hr APAP-challenged mice were measured. n=5. Experiment was repeated twice. (**d**) Comparison of hepatic GSH levels between WT and Nox2$^{-/-}$ mice. GSH levels in liver homogenates were determined at 0, 2, 6 and 48 hr after APAP challenge. n=3. Experiment was repeated twice. (**e**) The levels of H_2O_2 in the serum of WT/Gcsf$^{-/-}$ and Nox2$^{-/-}$/Gcsf$^{-/-}$ chimeras at 24 hr after APAP challenge were measured. n=5. Experiment was repeated twice. The data shown are means ± s.e.m (**P*<0.05, ***P*<0.01, ****P*<0.001). *P* values were calculated by one-way ANOVA (**b**) and two-tailed Student's *t*-test (**c**-**e**).

Supplementary Figure 6

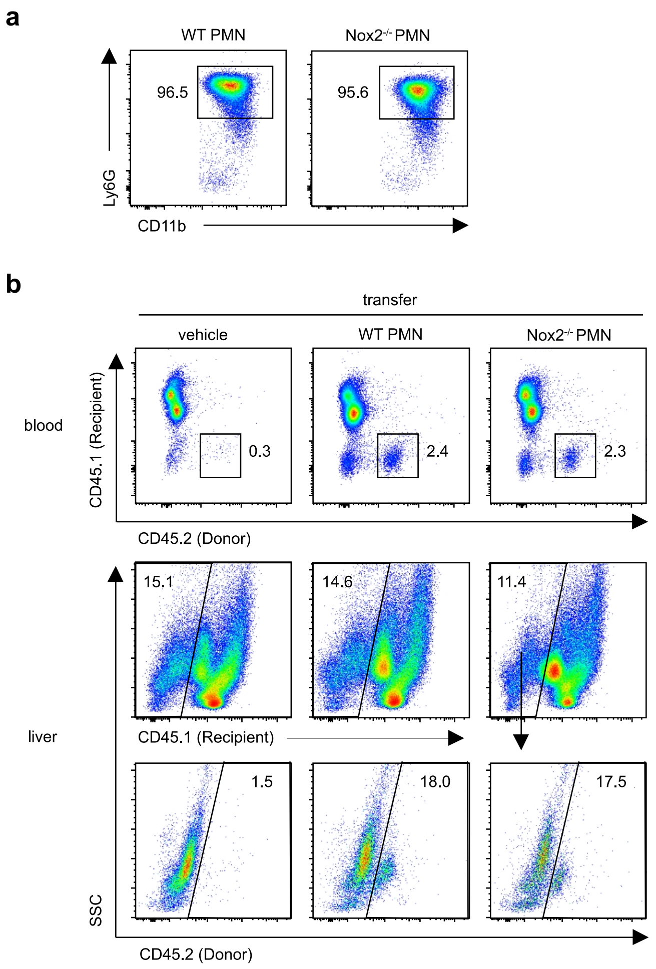

Supplementary Figure 6. Adoptive transfer of bone marrow-derived neutrophils.

(**a**) WT and Nox2$^{-/-}$ neutrophils were purified from bone marrow using Neutrophil Isolation Kit. Flow cytometric analysis of purified neutrophils before transfer. (**b**) Purified bone-marrow-derived neutrophils (CD45.2^{+}) were adoptively transferred into APAP-challenged CD45.1 mice. Three hours later, the percentage of donor neutrophils in the blood and liver of recipients was evaluated by flow cytometry. Experiment was repeated twice.

Supplementary Figure 7

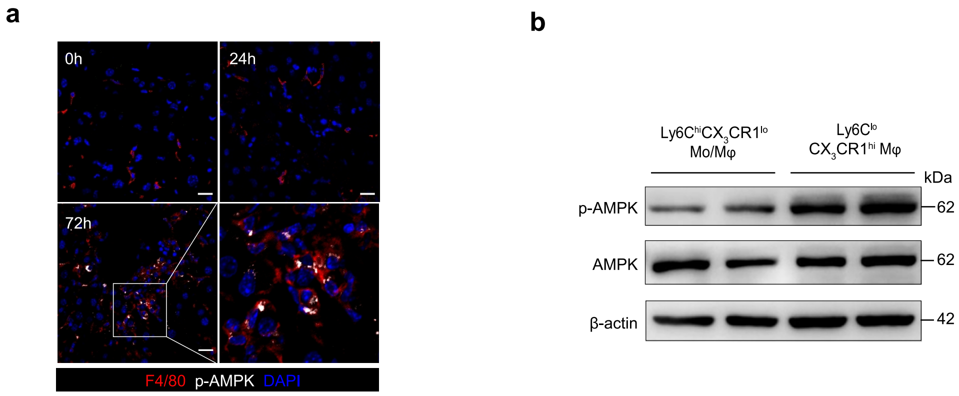

Supplementary Figure 7. Increased phosphorylation of AMPK in 72 hr Ly6C^{lo}CX$_3$CR1^{hi} macrophages.

(**a**) Confocal images from livers of untreated or APAP-challenged mice stained with anti-F4/80 and anti-phospho-AMPK (p-AMPK) antibodies. Nucleus were counterstained with DAPI. Scale bars, 20 μm. Images are representative of at least three independent experiments. (**b**) Western blot analysis for p-AMPK expression in 24 hr Ly6C^{hi}CX$_3$CR1^{lo} monocytes/macrophages and 72 hr Ly6C^{lo}CX$_3$CR1^{hi} macrophages. Experiment was repeated twice. Uncropped scans of western blots are shown in **Supplementary Fig. 8**.

Supplementary Figure 8

Related to Fig. 6a

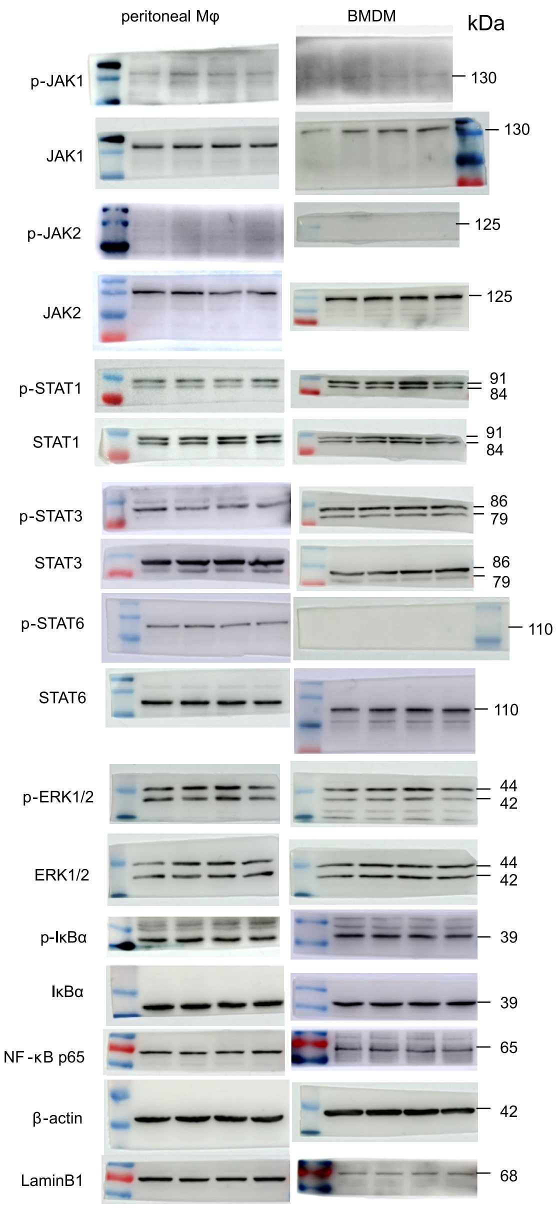

Related to Fig. 6b

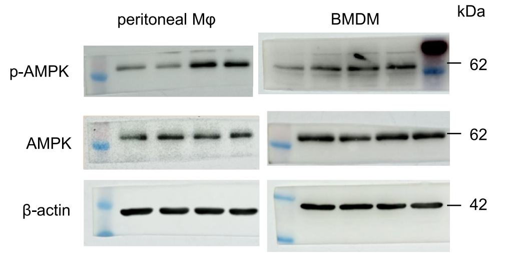

Related to Fig. 6c

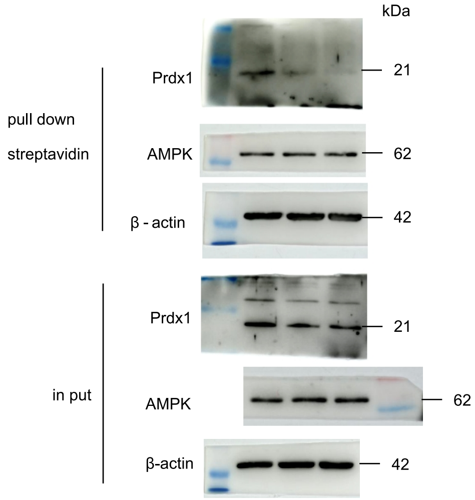

Related to Fig. 6e

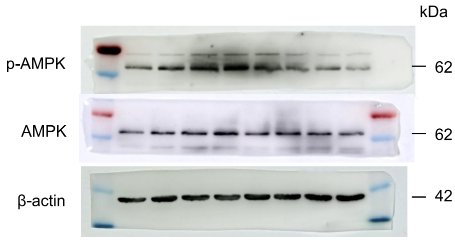

Related to Fig. 6f

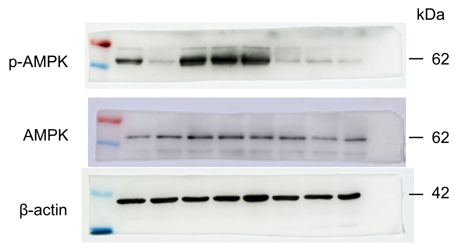

Related to Supplementary Fig. 5b

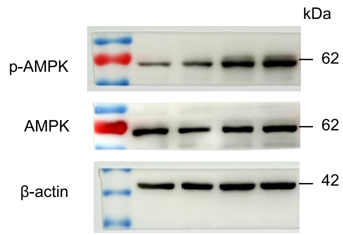

Supplementary Figure 8. Uncropped scans of western blots.

Supplementary Table 1. List of primers used for quantitative real-time PCR

Genes	Forward Primer	Reverse Primer
Gapdh	5'-AGGTCGGTGTGAACGGATTTG-3'	5'-TGTAGACCATGTAGTTGAGGTCA-3'
Hgf	5'-ACTTCTGCCGGTCCTGTTG-3'	5'-CCCCTGTTCCTGATACACCT-3'
Mrc1	5'-CTCTGTTCAGCTATTGGACGC-3'	5'-CGGAATTTCTGGGATTCAGCTTC-3'
Il1b	5'-TGTAATGAAAGACGGCACACC-3'	5'-TCTTCTTTGGGTATTGCTTGG-3'
Entpd1	5'-AAGGTGAAGAGATTTTGCTCCAA-3'	5'-TTTGTTCTGGGTCAGTCCCAC-3'
Cx3cr1	5'-CAGCATCGACCGGTACCTT-3'	5'-GCTGCACTGTCCGGTTGTT-3'
Cxcl10	5'-CCAAGTGCTGCCGTCATTTTC-3'	5'- GGCTCGCAGGGATGATTTCAA-3'
Fizz1	5'-CCCTCCACTGTAACGAAGACTC-3'	5'-CACACCCAGTAGCAGTCATCC-3'
Tgfb	5'-CTCCCGTGGCTTCTAGTGC-3'	5'-GCCTTAGTTTGGACAGGATCTG-3'
Cd36	5'-TTTCCTCTGACATTTGCAGGTCTA-3'	5'-AAAGGCATTGGCTGGAAGAA-3'
Il6	5'-TACCACTTCACAAGTCGGAGGC-3'	5'-CTGCAAGTGCATCATCGTTGTTC-3'
Nos2	5'-GGGCTGTCACGGAGATCA-3'	5'-CCATGATGGTCACATTCTGC-3'
Ym1	5'-CAGGGTAATGAGTGGGTTGG-3'	5'-CACGGCACCTCCTAAATTGT-3'
Il10	5'-GCTCTTACTGACTGGCATGAG-3'	5'-CGCAGCTCTAGGAGCATGTG-3'
