# Supplementary material for: Neutrophils promote the development of reparative macrophages mediated by ROS to orchestrate liver repair
Source: Nat Commun. 2019 Mar 6;10:1076. doi: 10.1038/s41467-019-09046-8 (PMC6403250; doi:10.1038/s41467-019-09046-8)
Supplement: Supplementary file 3 — Reporting Summary [file 41467_2019_9046_MOESM3_ESM.pdf]

nature research

Corresponding author(s): Li Tang

Reporting Summary

Nature Research wishes to improve the reproducibility of the work that we publish. This form provides structure for consistency and transparency in reporting. For further information on Nature Research policies, see Authors & Referees and the Editorial Policy Checklist.

Statistical parameters

When statistical analyses are reported, confirm that the following items are present in the relevant location (e.g. figure legend, table legend, main text, or Methods section).

n/a	Confirmed	
☐	☒	The exact sample size (*n*) for each experimental group/condition, given as a discrete number and unit of measurement
☐	☒	An indication of whether measurements were taken from distinct samples or whether the same sample was measured repeatedly
☐	☒	The statistical test(s) used AND whether they are one- or two-sided<br>*Only common tests should be described solely by name; describe more complex techniques in the Methods section.*
☒	☐	A description of all covariates tested
☐	☒	A description of any assumptions or corrections, such as tests of normality and adjustment for multiple comparisons
☐	☒	A full description of the statistics including central tendency (e.g. means) or other basic estimates (e.g. regression coefficient) AND variation (e.g. standard deviation) or associated estimates of uncertainty (e.g. confidence intervals)
☐	☒	For null hypothesis testing, the test statistic (e.g. *F*, *t*, *r*) with confidence intervals, effect sizes, degrees of freedom and *P* value noted<br>*Give P values as exact values whenever suitable.*
☒	☐	For Bayesian analysis, information on the choice of priors and Markov chain Monte Carlo settings
☒	☐	For hierarchical and complex designs, identification of the appropriate level for tests and full reporting of outcomes
☒	☐	Estimates of effect sizes (e.g. Cohen's *d*, Pearson's *r*), indicating how they were calculated
☐	☒	Clearly defined error bars<br>*State explicitly what error bars represent (e.g. SD, SE, CI)*

Our web collection on statistics for biologists may be useful.

Software and code

Policy information about availability of computer code

Data collection	BD FACSDiva Software Version 8.0.1 (Flow Cytometry), BioRad iCycler iQ5 Real-Time PCR detection system (qPCR)
Data analysis	FlowJo software (Flow Cytometry), BioRad iCycler iQ5 software (qPCR), ImageJ software Version 1.50i (Immunohistochemistry), Zeiss Zen software (Immunofluorescence), GraphPad Prism 5.0 (Statistics)

For manuscripts utilizing custom algorithms or software that are central to the research but not yet described in published literature, software must be made available to editors/reviewers upon request. We strongly encourage code deposition in a community repository (e.g. GitHub). See the Nature Research guidelines for submitting code & software for further information.

Data

Policy information about availability of data

All manuscripts must include a data availability statement. This statement should provide the following information, where applicable:

- Accession codes, unique identifiers, or web links for publicly available datasets
- A list of figures that have associated raw data
- A description of any restrictions on data availability

All the relevant data supporting the findings of this study are available within the article, or from the corresponding author on reasonable request.

Field-specific reporting

Please select the best fit for your research. If you are not sure, read the appropriate sections before making your selection.

☒ Life sciences ☐ Behavioural & social sciences ☐ Ecological, evolutionary & environmental sciences

For a reference copy of the document with all sections, see nature.com/authors/policies/ReportingSummary-flat.pdf

Life sciences study design

All studies must disclose on these points even when the disclosure is negative.

Sample size	Sample sizes were estimated on the basis of sample availability and no statistical methods were used to predetermine sample size.
Data exclusions	No exclusion criteria were applied, and all samples were included in data analysis.
Replication	All experiments were repeated with similar results as stated in the Figure Legends.
Randomization	No formal randomization was used and animals were unbiasedly assigned into different treatment groups.
Blinding	Group allocation and outcome assessment was performed in a blinded manner.

Reporting for specific materials, systems and methods

Materials & experimental systems

n/a	Involved in the study	
☒	☐	Unique biological materials
☐	☒	Antibodies
☒	☐	Eukaryotic cell lines
☒	☐	Palaeontology
☐	☒	Animals and other organisms
☒	☐	Human research participants

Methods

n/a	Involved in the study	
☒	☐	ChIP-seq
☐	☒	Flow cytometry
☒	☐	MRI-based neuroimaging

Antibodies

Antibodies used

Flow Cytometry:
CD45-PerCP/Cy5.5 or APC/Cy7 (eBioscience, cat# 450451, or Biolegend, cat# 103115, 30-F11)
Ly6G-BV421 or PECy7 (BD Biosciences, cat# 562727 or 560601, 1A8)
F4/80-PE or FITC (eBioscience, cat# 124801 or 114801, BM8)
CD11b-APC (eBioscience, cat# 170112, M1/70)
CD3-eFluor 450 (eBioscience, cat# 480032, 17A2)
CD19-FITC (eBioscience, cat# 110193, eBio1D3)
CD49b (DX5)-PE (eBioscience, cat# 125971, DX5)
γδTCR-APC (eBioscience, cat# 118115, GL3)
CD11c-PE (eBioscience, cat# 117347, N418)
Siglec-F-BV421 (BD Biosciences, cat# 565934, E50-2440)
Ly6C-PECy7 or AF488 (eBioscience, cat# 255932 or cat# 535932, HK1.4)
CD64-PE (eBioscience, cat# 120641, X54-5/7.1)
MerTK-PE (eBioscience, cat# 125751, DS5MMER)
CX3CR1-PE (Biolegend, cat# 149005, SA011F11)
Gr-1-FITC (eBioscience, cat# 115931, RB6-8C5)
MHC-II-FITC (eBioscience, cat# 115321, M5/114.15.2)
CD45.1-BV421 (eBioscience, cat# 110731, A20)
CD45.2-PE (eBioscience, cat# 125941, 104)

Western Blot:
p-JAK1 (Cell Signaling Technology, cat# 74129)
JAK1 (Cell Signaling Technology, cat# 3344)
p-JAK2 (Cell Signaling Technology, cat# 8082)
JAK2 (Cell Signaling Technology, cat# 3230)

p-STAT1 (Cell Signaling Technology, cat# 7649)
STAT1 (Cell Signaling Technology, cat# 9172)
p-STAT3 (Cell Signaling Technology, cat# 9134)
STAT3 (Cell Signaling Technology, cat# 4904)
p-STAT6 (Cell Signaling Technology, cat# 56554)
STAT6 (Cell Signaling Technology, cat# 5397)
p-ERK1/2 (Cell Signaling Technology, cat# 9101)
ERK1/2 (Cell Signaling Technology, cat# 3230)
p-IκBα (Cell Signaling Technology, cat# 2859)
IκBα (Cell Signaling Technology, cat# 4814)
NF-κB p65 (Cell Signaling Technology, cat# 8242)
Lamin B1 (Cell Signaling Technology, cat# 13435)
p-AMPKα (Cell Signaling Technology, cat# 2535)
AMPKα (Cell Signaling Technology, cat# 2532)
Prdx1 (Abcam, cat# ab15571)
β-actin (Sigma Aldrich, cat# A5441)

Immunofluorescence
F4/80 (Abcam, cat# ab6640, CI:A3-1)
p-AMPKα (Thr172) antibody (Cell Signaling Technology, cat# 2535, 40H9)

Validation: All antibodies used were validated in the literature or by suppliers.

Animals and other organisms

Policy information about studies involving animals; ARRIVE guidelines recommended for reporting animal research

Laboratory animals: All the mouse strains used were on a C57BL/6 background. Male mice and their littermate control between six- to eight-week-old were used.

Wild animals: The study did not involve wild animals.

Field-collected samples: The study did not involve field-collected samples.

Flow Cytometry

Plots

Confirm that:

☒ The axis labels state the marker and fluorochrome used (e.g. CD4-FITC).

☒ The axis scales are clearly visible. Include numbers along axes only for bottom left plot of group (a 'group' is an analysis of identical markers).

☒ All plots are contour plots with outliers or pseudocolor plots.

☒ A numerical value for number of cells or percentage (with statistics) is provided.

Methodology

Sample preparation: The information is available in Methods, "Primary cell isolation" subsection.

Instrument: LSRFortessa cell analyzer (BD Biosciences) and FACSAria III (BD Biosciences).

Software: BD FACSDiva Software was used to collect the flow cytometry data; FlowJo software was used to analyze the data.

Cell population abundance: Post-sort purity > 95% as described in Methods.

Gating strategy: All data were first gated according to: FSC/SSC (remove debris) -> FCS Height/FCS Area(remove dublets). The follwing gating strategies were provided in individual figure legends.

☒ Tick this box to confirm that a figure exemplifying the gating strategy is provided in the Supplementary Information.
